# Effect of Substrate Surface Roughening on the Capacitance and Cycling Stability of Ni(OH)_2_ Nanoarray Films

**DOI:** 10.1038/s41598-019-53365-1

**Published:** 2019-11-14

**Authors:** Adulphan Pimsawat, Apishok Tangtrakarn, Nutsupa Pimsawat, Sujittra Daengsakul

**Affiliations:** 1 Department of Physics, Faculty of Science, Khon Kaen University, Khon Kaen, 40002 Thailand; 2 Institute of Nanomaterials Research and Innovation for Energy (IN-RIE), NANOTEC-KKU RNN on Nanomaterials Research and Innovation for Energy, Khon Kaen University, Khon Kaen, 40002 Thailand; 3 Institute of Nanomaterials Research and Innovation for Energy (IN-RIE), Khon Kaen University, Khon Kaen, 40002 Thailand; 4 Department of Chemical Engineering, Faculty of Engineering, Khon Kaen University, Khon Kaen, 40002 Thailand

**Keywords:** Supercapacitors, Synthesis and processing, Surfaces, interfaces and thin films

## Abstract

The effect of substrate surface roughening on the capacitance of Ni(OH)_2_/NiOOH nanowall array samples produced via chemical bath deposition for 2, 4, 6, 24 and 48 h on an as-received stainless steel substrate and the same substrate after sandblasting has been investigated. Symmetric cells were subjected to 120,000 charge-discharge cycles to access changes in their capacitance. Specific capacitances were derived from cyclic voltammetry and charge-discharge cycling under a three electrode setup. Substrate roughening significantly increases the capacitance of symmetric cells and film stability since film exfoliation does not occur to the same degree as on the as-received substrate. Interestingly, films deposited on a roughened substrate for 6, 24 and 48 h also exhibit self-recovery of capacitance, which could be related to an electrodissolution-electrodeposition effect. With the use of a roughened substrate, the thinnest film gives the highest specific capacitance, 1456 F g^−1^, whilst the thickest one shows the highest areal capacitance, 235 mF cm^−2^, after 20,000 cycles. These results reveal the promise of surface roughening toward increasing the capacitance and stability of Ni(OH)_2_/NiOOH films.

## Introduction

A supercapacitor is an electrochemical device that can store and release electricity at a high power density. Unlike a battery, supercapacitors can handle unstable electrical power, so they have been widely used to directly collect energy from other power generators such as wind turbines, solar cells and regenerative brakes^1–3^. Furthermore, supercapacitors can be set to discharge at various high-current rates, hence they have also been selectively employed to start high-torque motors^4^. There are three common types of capacitors which are electrical double layer capacitors, pseudocapacitors and hybrid capacitors. Electrical double-layer capacitors (EDLC) and pseudocapacitors rely on the transfer of electrostatic charge at the surface of an electrode and on electron exchange reaction/phase change of active materials, respectively^5^. Combining the advantages of EDLCs and pseudocapacitors creates a hybrid capacitor type.

Pseudocapacitors are interesting because they have high specific capacitance values, especially those made of transition metal hydroxide/oxyhydroxides such as nickel and cobalt^6^. Nickel hydroxide/oxyhydroxide based materials can be used to fabricate promising electrodes for supercapacitor applications because their nanostructures can be prepared by a facile chemical bath deposition^7–9^. In this study, films were prepared following a well-established precipitation technique^8,9^ to create nickel hydroxide/oxyhydroxide films, except that the nickel sulfate concentration in this case was reduced by half allowing precipitation to proceed more slowly than in other studies. The temperature in this study was maintained at ~30 °C.

In addition to a high specific capacity, long-term stability of an electrode material in terms of capacity change and film exfoliation should be investigated. Extending the cycling lifetime of the electrode should be done by increasing substrate roughness as film adhesion to the substrate could be improved^10^. Stainless steel (SS) sheets can be used as a substrate as well as a current collector for a supercapacitor. SS sheets can be roughened by one of several methods. They can be sandblasted and subsequently subjected to a mild acid etching^11,12^, anodization and subsequent heat treatment^13^, strong acid etching^14^, acid etching^15^ or anodization^16^. It is notable that the materials examined in these reports were not nickel hydroxide/oxyhydroxide films and their films were not prepared by precipitation method. Additionally, the effects of substrate roughening on the capacitance and long term stability of films were not studied. Herein, a sandblasted SS sheet (R) was compared to an as-received sheet (S) to elucidate the effect of substrate roughening (without subsequent acid/base treatment) on the capacitance and long term stability of precipitated Ni(OH)_2_/NiOOH films with various thicknesses after being subjected to 120,000 cycles. Interestingly, according to our observations, there is no published report that shows a capacity result for a precipitated Ni(OH)_2_/NiOOH film beyond 100,000 cycles. Furthermore, self-electrodeposition/electrodissolution of the film materials was investigated in the current study by setting the charging current higher than the discharge current. Notably, this also helped to reduce the time required for experimentation. If such phenomena exist, the morphology and capacities of films on one side of symmetrical electrode could be different from those of films on the opposite side. It is speculated that electrodeposition of a film material could predominately occur on the negative electrode (R1, S1) (assigned at the first charging step) and the electro-dissolution of the film could primarily occur at the positive electrode (R2, S2). According to our best knowledge, this phenomenon is induced by long charge and discharge cycling also and has never before been reported in a supercapacitor system. This phenomenon could impact the film cycling stability in the long term.

## Results and Discussion

### Field emission scanning electron microscopy (FE-SEM)

**Figure 1:**
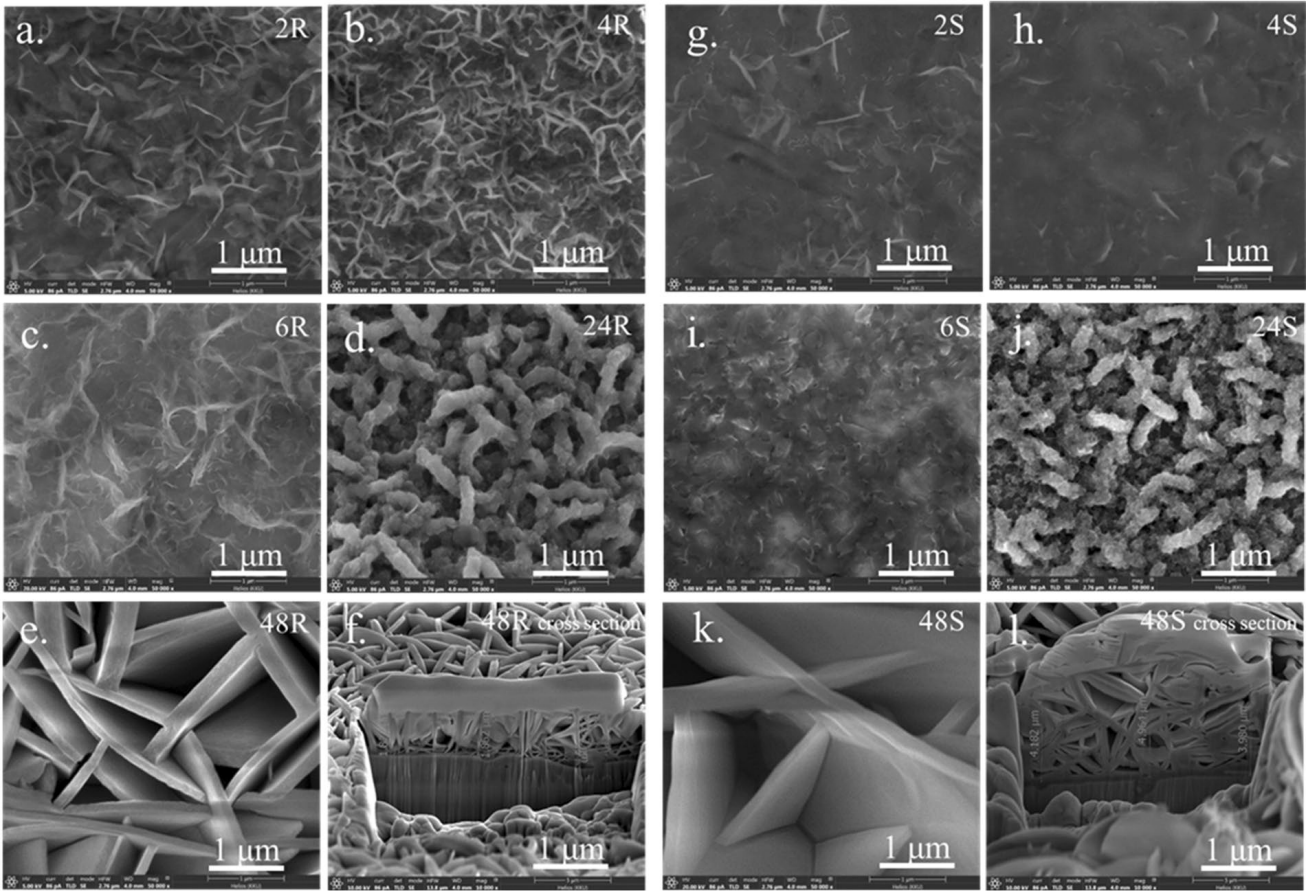
FE-SEM images of films (**a**) 2 R (**b**) 4 R (**c**) 6 R (**d**) 24 R (**e**) 48 R (**f**) cross-section of 48 R (**g**) 2 S (**h**) 4 S (**i**) 6 S (**j**) 24 S (**k**) 48 S (**l**) cross-section of 48 S.

**Figure 2:**
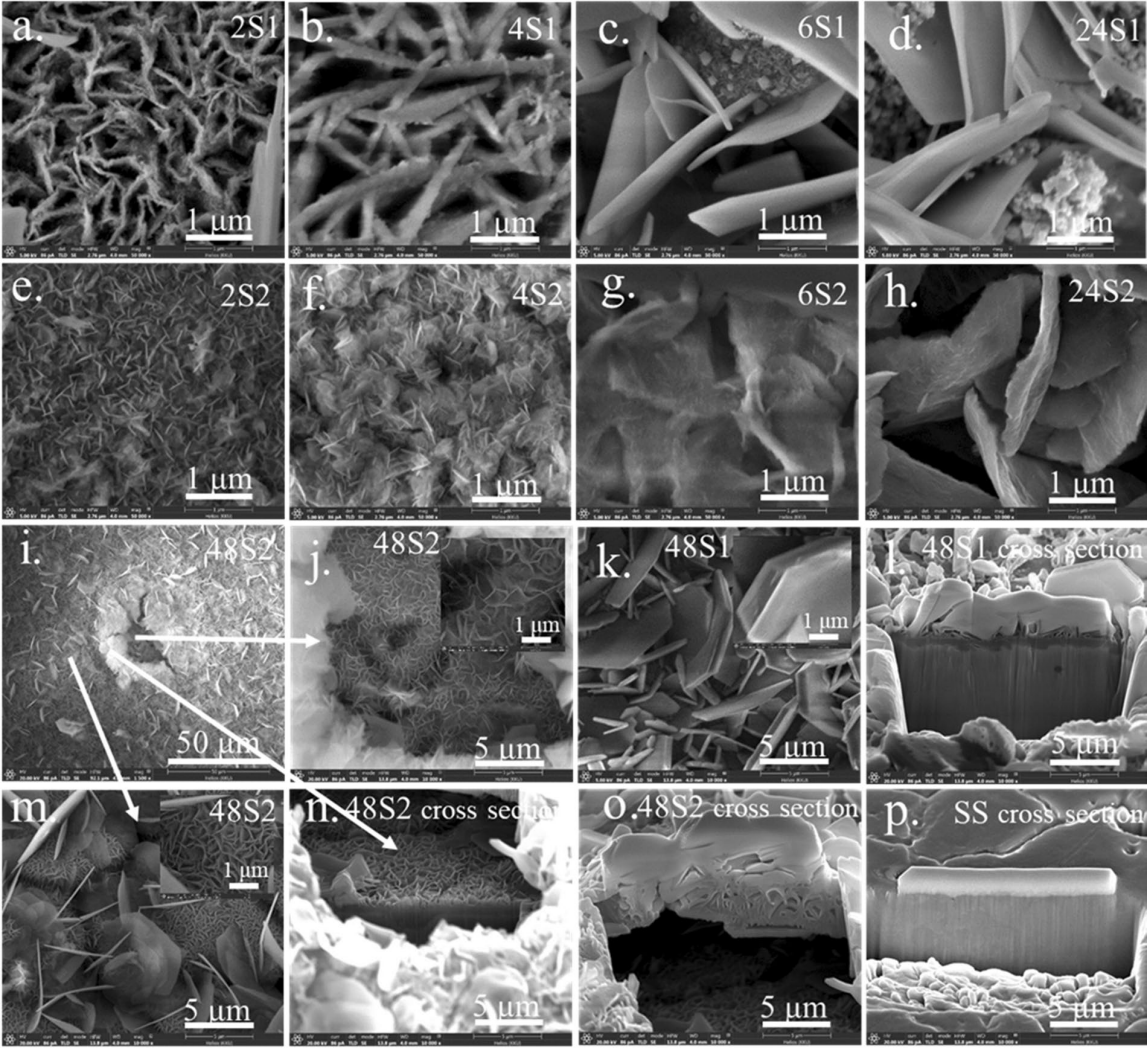
FE-SEM image of films (**a**) 2S1 (**b**) 4S1 (**c**) 6S1 (**d**) 24S1 (**e**) 2S2 (**f**) 4S2 (**g**) 6S2 (**h**) 24S2 (**i**) 48S2 (**j**) 48S2 inset hole (**k**) 48S1 (**l**) cross-section of 48S1. (**m**) 48S2 inset uncracked films (**n**) under cross-section of 48S2 (**o**) over cross-section of 48S2 (**p**) cross-section of as receive stainless steel.

**Figure 3:**
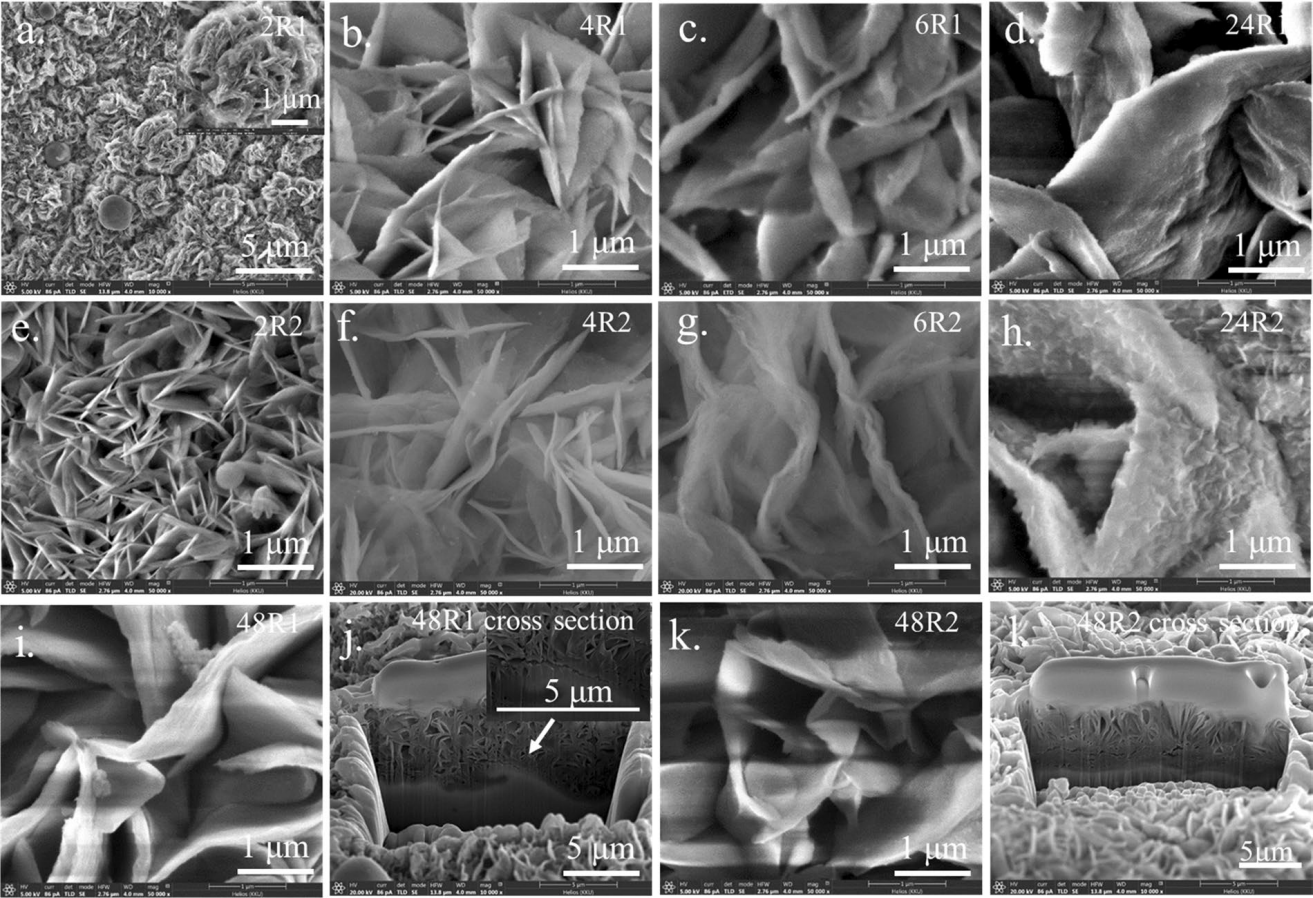
FE-SEM images of films (**a**) 2R1, (**b**) 4R1, (**c**) 6R1, (**d**) 24R1, (**e**) 2R2, (**f**) 4R2, (**g**) 6R2, (**h**) 24R2, (**i**) 48 R1, (**j**) cross-section of 48R1, (**k**) 48R2, (**l**) cross-section of 48R2.

**Figure 4:**
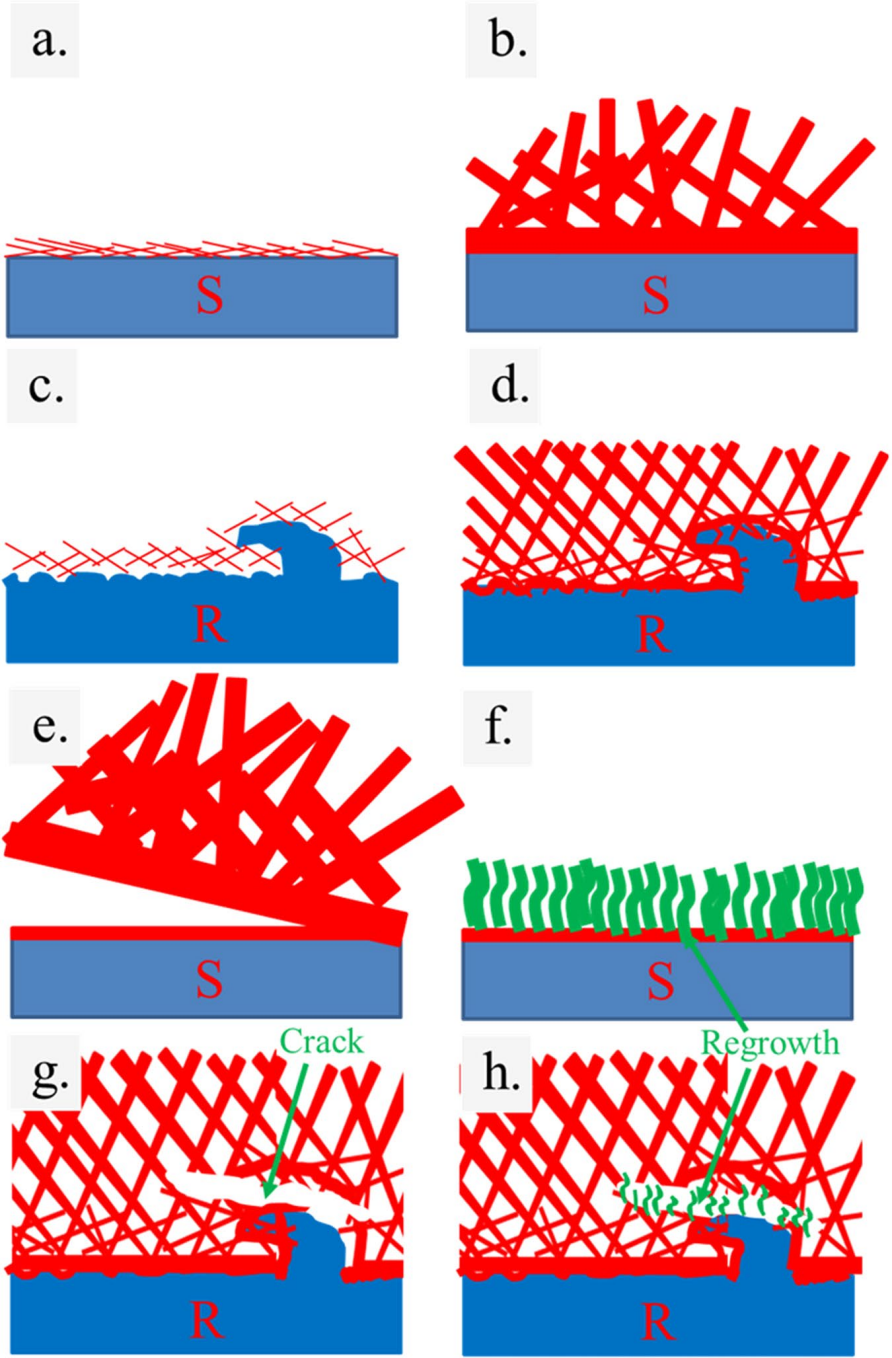
Schematic drawings to describe how films are formed on (**a**,**b**) as-received substrate and (**c**,**d**) roughened substrate, as well as a description of crack propagation (**e**) and regrowth (**f**) occurs in a film on a smooth substrate and on a roughen substrate (**g**,**h**).

Figures 1 and 2 show the morphologies of representative as-deposited films on the smooth and rough substrates as a function of deposition time, respectively. Initially, the precipitates form 2D nanosheets that subsequently aggregate and connect to one another, resulting in a network architecture on the substrates^17,18^. However, some precipitates may not integrate well onto the main wall resulting in stain-like precipitates covering the nanowall surfaces. This noticeably occurs with films grown on the as-received substrate for 2, 4 and 6 h. A roughened substrate has many uneven surfaces, so incoming precipitate can readily latch onto pre-formed nanosheets (see Fig. 4a) rather than the as-received substrate (Fig. 4c). For this reason, incorporation of precipitates onto the nanostructure is improved (less stain-like surface was observed) for a rougher surface compared to that of the film on the as-received substrate. For both types of substrates, nanostructures became thicker and taller with increasing time (see Fig. 4b, 4d). The wall surface of films on both types of substrates became smoother after 48 h of precipitation, which implies a significant reduction of the growth rate due to depletion of precursor ions from the deposition bath over such a long time. This results in a merging of nanowalls into smooth wall surfaces (Fig. 1e, 1k). Cross-sections of 48 S and 48R using Focused Ion Beam-Scanning Electron Microscopy (FIB-SEM) are shown in Fig. 1f and 1l, respectively. Notably, numerous cells with thinner walls on the substrates exist in 48 R but not 48 S, which supports the proposed precipitation mechanism. This difference in the contact layer substructure may contribute to improved film stability after long cycling periods. Film morphologies after 120,000 cycles are shown in Figs 2 and 3. The change in morphology according to electrodeposition was investigated after such long cycling. It is notable that extra NiSO_4_ from an external source was not added. Moreover, since the charging and discharging currents are different, the positive and negative electrodes were separately characterized.

If electrodeposition occurs, the crystals should grow larger during charge and discharge cycling. In order to compare the size of the wall before and after cycling, images having a one-micron scale (shown as an inset image for some samples) were used. The morphologies shown in Figs 2 and 3 compared to those of Fig. 1 support the existence of an electrodeposition phenomenon since the height and thickness of walls became greater for all films deposited for 2, 4, 6, 24 h after cycling. Though the wall thickness of 48 R1 and 48R2 appears smaller than that of 48R, it is clear from the cross-sectional view that there is additional growth of film in the vertical direction. So, the wall became thinner at the top (Figs 1f, 2j, 2l). In the case that the height and width of the wall are actually reduced after cycling, the film already cracks or exfoliates in those particular areas. This leaves remnant materials close to exposed substrate as shown in Fig. 2j, 2n. The exposed area could experience some extra film growth because the walls have the characteristics of film-growth arrays. Generally, thick films grown on a smooth substrate (24S1, 24S2, 48S1 and 48S2) present exfoliation that can be visually observed. Though thick films on a roughened substrate (6R1, 6R2, 24R1, 24R2, 48R1 and 48R2) have no exfoliation issues as observed without magnification, some minor cracks could still be found using FE-SEM (inset Fig. 3j). This confirms the benefits of using a roughened substrate. It enables the film to be less prone to exfoliation, as illustrated in Fig. 4g and 4h compared film growth on a smooth substrate (Fig. 4e). On a roughened substrate, nucleated cracks have difficulty propagating through the film region in contact with the rough substrate for two reasons. First, the area adjacent to the substrate contains numerous small cell voids that are surrounded by nanowalls (Fig. 1f, 4c, 4d). Second, it could also be hard for the film to be pulled from the hook-like edges on the substrate surface created by sandblasting. From FE-SEM imagery, crack propagation typically occurs above the hook-like edges on the substrate surface (Fig. 4h). Though the film is not an elastic polymer that effectively anchors itself in the grooves of a roughened substrate^10^, it is likely that the combination of the two proposed mechanisms effectively increases film stability. Film stability as well as film dissolution/regrowth was investigated at various rates of charging and discharging.

**Figure 5:**
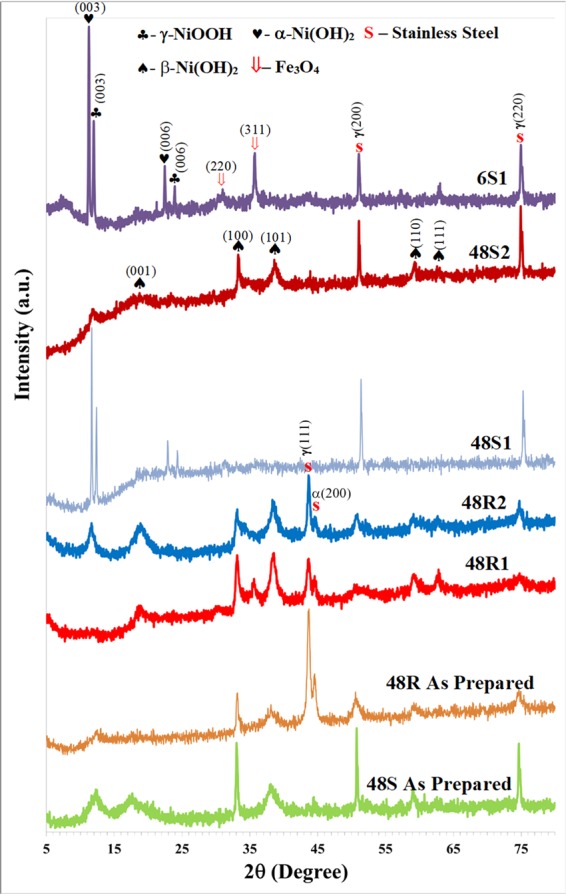
XRD patterns of as-prepared nickel hydroxide/oxyhydroxide films (48R and 48S) and after 120,000 cycling (6S1, 48S1, 48S2, 48R1 and 48R2).

Since the discharge current (1 mA) is smaller than the charging current (4 mA) in a two electrode test, a negative electrode assigned during charging (R1, S1) could gain more materials from the positive side (R2, S2) than is lost to the opposite side and vice versa. Unfortunately, a significant difference between morphology of R1 and R2 attributable to electrodissolution/electrodeposition cannot be clearly distinguished. As previously mentioned, crystal growth was observed when the R1 and R2 samples were compared to R whilst the S1 set shows a clearer crystal growth effect on the 1st side. These films were deposited for 2, 4, 6 h. This means that electrodissolution/electrodeposition actually takes place. It is notable that 24 S and 48 S experience multiple film exfoliation and growth events, so comparison between S1 and S2 after long cycling might not be insightful. Nevertheless, from visual observation, 24S2 and 48S2 are exfoliated earlier than 24S1 and 48S1, respectively. Additionally, from FE-SEM images, large crystals are present in 24S1 and 48S1 (Fig. 3d, 3k). Abnormally large crystals are more frequently observed on the surfaces of S1 samples than those of S2 sets. This confirms the existence of electrodeposition and electroetching as Ni ions come from the dissolution of Ni-based films during cycling. A reason why an electrodeposition/electroetching effect becomes more prominent for films on a smooth substrate is because these films tend to break off at an early stage of cycling (less than 1,000 cycles). This provides an increased electrical field and subsequently leads to the regrowth of the crystals on the both sides, especially on the negative electrode (Fig. 2k). Exfoliation of films on the roughened substrate was seldom observed. Therefore, the electrical field is less than that of a film grown on a smooth substrate. Moreover, the number of Ni ions that contribute to reconstruction of nanowalls is less when a roughened substrate is used because their films rarely separate from the substrate and dissolve in the electrolyte. As the experiment was done over the course of a year, dissolution of film would take place. Crystal growth, which comes with improved crystallinity, was confirmed by XRD analysis. The diffraction patterns used to check the crystallinity of the samples on a both substrates are shown in Fig. 5. Strong diffraction peaks from large crystals are expected for those films grown on a smooth substrate, especially on the negative electrode.

### X-ray diffraction (XRD)

The XRD patterns of selected samples in Fig. 5 (a) reveal that as films were deposited onto roughened and smooth substrates, they primarily consisted of mixed phases of α-Ni(OH)_2_ (JCPDS 38-0715), β-Ni(OH)_2_ (JCPDS 14-0117) and γ-NiOOH (JCPDS 06-0075). Peaks related to a γ-NiOOH phase were not as clearly observed in the diffraction patterns of the as-prepared samples compared to those of previous reports^19,20^. The γ-NiOOH phase, which was below the minimal detection limit of XRD, should still exist since persulfate ions can readily react with Ni(OH)_2_ in the precipitation bath to form a γ-NiOOH phase^9,20^. Less NiOOH phase is observed than in previous studies because the solution in the precipitation bath is stirred only at the initial stage during ammonia addition. Therefore, the oxidation reaction between Ni(OH)_2_ and K_2_S_2_O_8_ at a surface of the vertically-placed substrate could be less prevalent than competing reactions that occur at the bottom of the precipitation bath. Actually, precipitates collected from the bottom of the beakers contained a γ-NiOOH phase (data not shown). The XRD phases belong to films of R2 and S2, which are similar to those of as-prepared films, but are different from R1 and S1. Furthermore, the R1 and S1 phases also differ from each other. The presence of a particular phase is independent of deposition time, therefore only certain XRD spectra are shown here. The S1 film contains α-Ni(OH)_2_ and γ-NiOOH, whilst that of R1 has only a β-Ni(OH)_2_ phase. Strong diffraction peaks ascribed to α-Ni(OH)_2_ and γ-NiOOH are clearly visible in the 6S1 and 48S1 samples, which corresponds well to the enlarged crystals observed using FE-SEM. This enlarged crystal from crystal regrowth could be responsible for the slow phase change back to β-Ni(OH)_2_ in films grown on a smooth substrate. It is notable that signature peaks of stainless steel (JCPDS 34-0396), which are γ(111) and α(200), appear only after the substrates were polished. The α(200) peak appears due to surface roughening and could induce formation of martensite^21^. The phase corresponding to γ(111), which normally exists in stainless steel material, disappears in the as-received substrate due to different preferred orientations induced by the manufacturer’s processing steps. Interestingly, the substrate on the positive electrode experiences a degree of corrosion as Fe_3_O_4_ peaks (JCPDS 19-0629) are clearly present only on the negative electrode. Development of Fe_3_O_4_ clearly indicates electrodeposition that predominantly occurs on the negative electrode, while electroetching of substrate take places on the positive electrode. The explanation for Fe_3_O_4_ formation was previously given in reports involving electrochemical synthesis of nano Fe_3_O_4_ to induce corrosion of a pure ion film^22^. Their average particle size was in the range of 10–30 nm, depending on the electrode distance and current density. In our case, the particle size of Fe_3_O_4_ is approximated from Scherer’s equation as ∼20 nm.

### Symmetrical two electrode capacitance (CD-2) performance

**Figure 6:**
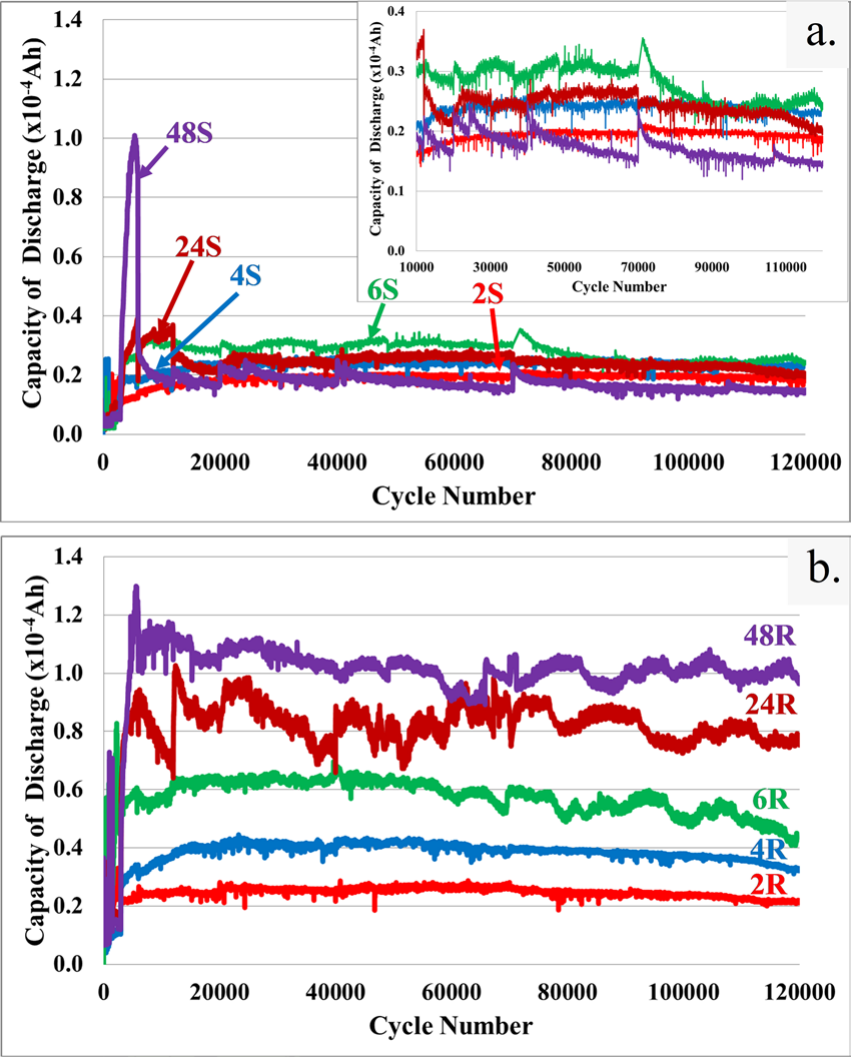
Capacity of discharge during cycling for films (**a**) S and (**b**) R.

The discharge capacitance value of films grown on a smooth substrate was measured under a two electrode setup and the results are shown in Fig. 6a and 6b. It is notable that an abrupt discrete increase or decrease in capacitance (one data point) is due to instrumental errors. The relationship between the cycle number and discharge capacity can be divided into two stages. In the first stage, the discharge capacity is increasing with the number of cycles to a transition point that separates the first and second stages. Increasing capacitance during the first stage is only partially related to the activation process, which commonly occurs at 100–1000 cycles for Ni(OH)_2_^20,23^. For roughened substrates, such a transition point for thicker films (24 S, 48 S) occurs at ∼5000–7000 cycles, whilst those of thinner films (2 S, 4 S, 6 S) occur at ∼10,000–20,000 cycles. The longer transition time observed in this study over that of other published reports^20,23^ could be due to an additional mechanism involving the regrowth or etching of film, which prevails at the negative and positive electrodes, respectively. The regrowth mechanism is illustrated in Fig. 4f,h. At the second stage, the capacitances of the thinner films on a smooth substrate slightly fluctuate around an average capacitance value, whilst those of thicker films decrease abruptly due to film exfoliation. Nevertheless, the capacitances of thicker films (especially for 48 S) can also randomly increase to a slight degree afterwards. This implies that some portions of the film could still stay active though other parts may exfoliate and that electrodeposition facilitated regrowth of nanowalls can occur. Direct regrowth of film on a smooth substrate where exfoliation occurs in Fig. 2n occurs by the mechanism shown in Fig. 4f.

For films grown on a roughened substrate, capacitance enhancement was observed in the first stage, whilst random switching between capacity fading and growth was prevalent in the second stage. Nevertheless, their capacitances eventually slowly fall off at certain high cycle numbers. Random increases of capacitance due to a self-healing mechanism after a capacity fading were clearly observed for thicker films (6 R, 24 R and 48 R). Recovery of capacitance values should be related to film regrowth that helps fill local cracks as illustrated in Fig. 4h. Interestingly, the difference in the smoothness of the discharge capacity curve of 24 R and 48 R could be related to the faster film healing of 48 R compared to that of 24 R since 48 R has more dissolved ions in the electrolyte. This could facilitate quicker regrowth of films over the damaged areas. However, the discharge capacity of 6 R is smoother than that of 24 R, especially for cycle numbers less than 60,000 because the capacity of 6 R is lower and crack propagation less extensive. Yet stronger fluctuation in the capacity value of 6 R was clearly observed for cycle numbers greater than 60,000 due to the increasing thickness and height of 6 R films, which might eventually induce the crack and regrowth phenomenon. Charge-discharge curves are presented in Fig. S1a and S1b. These curves show the typical patterns of a supercapacitor. Apart from charge-discharge measurements based on a two electrode setup, additional specific capacitance (F g^−1^) and area-specific capacitance (F cm^−2^) using a three electrode setup have previously been reported. Their values are higher than those of a two electrode configuration. In the next section the average maximum specific capacitance obtained from CV (CV-3) and CD (CD-3) under a three electrode setup was determined and compared with previous reports.

### Cyclic voltammetry (CV) on three electrode setup

**Figure 7:**
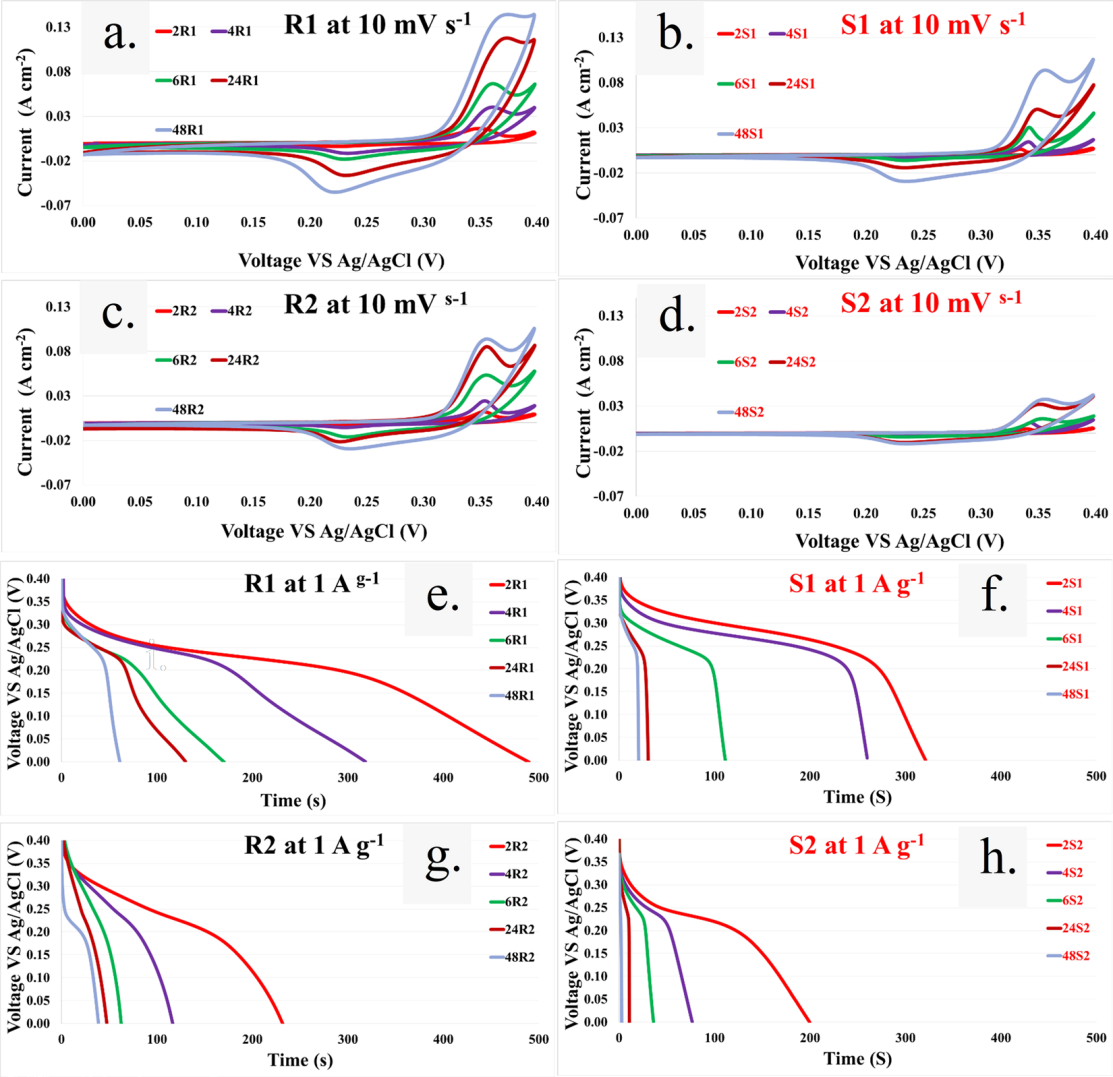
CV curves of (**a**) R1, (**b**) S1, (**c**) R2, (**d**) S2, and discharge curves of (**e**) R1, (**f**) S1, (**g**) R2, and (**h**) S2 after 20,000 cycles.

The electrochemical properties and the specific capacitance of a Ni(OH)_2_ electrode can be derived from cyclic voltammetry measurements. The CV curves (Fig. 7a, 7d) of samples after being cycled 20,000 times in a two electrode setup show a characteristic pseudo-capacitator behavior with redox peaks corresponding to Faradic reactions^9,20^:

1 $${\rm{Ni}}{({\rm{OH}})}_{2}+{{\rm{OH}}}^{-}\underset{{\rm{Discharge}}}{\overset{{\rm{Charge}}}{\rightleftharpoons }}{\rm{NiOOH}}+{{\rm{H}}}_{2}{\rm{O}}+{{\rm{e}}}^{-}$$

**Figure 8:**
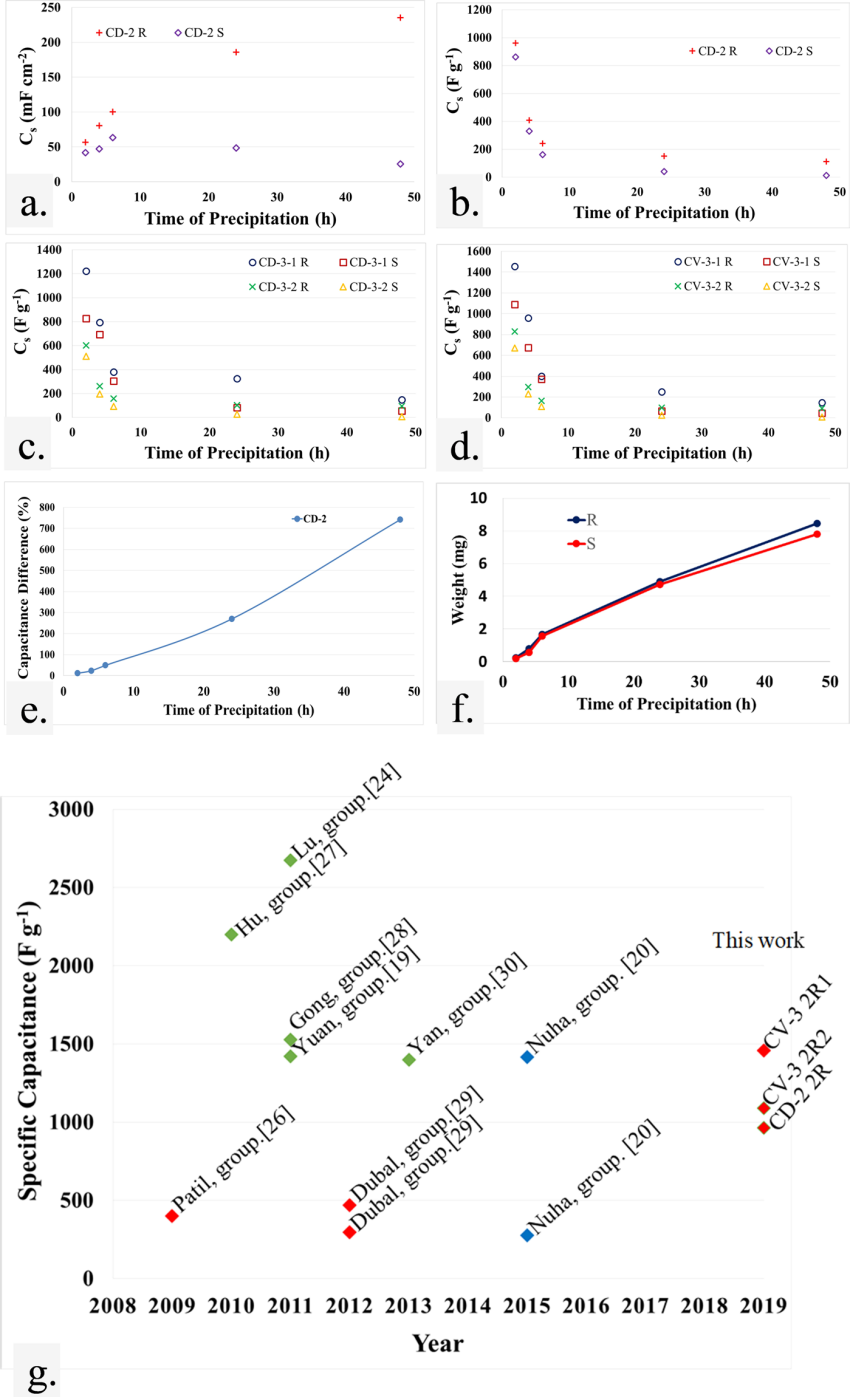
Graph of (**a**) area-C_s_ as a function of deposition time, (**b**) C_s_ as a function of deposition time, (**c**) C_s_ as a function of deposition time calculated from CD-3, (**d**) C_s_ as a function of deposition time calculated from CV-3, (**e**) area-C_s_ difference as a function of deposition time, (**f**) weight as a function of deposition time, and (**g**), a collection of published specific capacitance values for Ni(OH)_2_/NiOOH electrodes prepared by chemical bath deposition, hydrothermal and solvothermal methods. Red, blue and green colors denote stainless steel, fibrous carbon fabric and nickel foam substrates, respectively.

With the same precipitation time for all films, the absolute current densities (A cm^−2^) of the redox peaks of negative electrodes are higher than those of their positive counterparts. Additionally, increasing the precipitation time results in stronger redox reactions in all cases because of increasingly active materials. The specific capacitance values of each electrode derived from CV measurements (CV-3) are shown in Fig. 8d.

### Galvanostatic charge-discharge based on a three electrode setup

Figure 7e–h show typical discharge curves of each electrode tested in 6 M KOH at a constant load current of 1 A g^−1^. The curves show three different stages of discharge potential variation^24^. The potential abruptly drops due to the internal resistance in the initial stage. Then, the potential drops linearly with increasing time during the second stage. This indicates a double layer capacitance characteristic that corresponds to the charge separation at the interface between the electrode and electrolyte. The relationship between the potential and time then becomes nonlinear in the final stage, which is related to a typical pseudo-capacitance behavior stemming from redox reactions, electrochemical adsorption/desorption or ion intercalation. The discharge curves of films grown on both types of substrates exhibit pseudo-capacitator behavior, which agrees well with the CV results. The specific capacitance values of each electrode derived from galvanostatic charge-discharge in a three electrode setup (CD-3) are shown in Fig. 8c.

To study the substrate roughness effect, the relationship between precipitation time and the average C_s_ value from a two electrode setup was investigated (Fig. 8a, 8b). The differences (%) in C_s_ of films are presented in Fig. 8e. The average capacitance value of the negative and positive electrodes determined from a three electrode setup are compared in Fig. 8c and 8d. From Fig. 8a, the area specific capacitance of films grown on the roughened substrate increases with the deposition time. However, if a smooth substrate is used, C_s_ increases with time only for thinner films (2, 4 and 6 h). Thicker films (24 h, 48 h) grown on the smooth substrate extensively exfoliate, resulting in decreased C_s_ area with increasing time. For the same precipitation time, the C_s_ area of films on the roughened substrate is higher than that of films on a smooth substrate. Furthermore, differences in C_s_ (Fig. 8e) also increase with precipitation time. For thinner films, such differences arise since the surfaces of film grown on a roughened substrate are more compact than those on a smooth substrate. For thicker films, further differences arise due to exfoliation of film grown on the smooth substrate, which can be visually observed.

If the specific capacitance in units of Farad per effective mass is considered, the mass-related C_s_ is reduced with deposition time. This means that capacitance enhancement from additional mass does not grow due to the increasing divisor value which is the effective film mass. It may be more useful to consider C_s_ with the units, mF cm^−2^, because real-world practice involves application of films over large substrate areas. However, C_s_ in the units of F g^−1^ is typically reported as reducing the mass of active material can increase the reported C_s_ value. Nevertheless, errors in weight measurement lead to erroneous results^25^. An average deposited film mass as a function of time are shown in Fig. 8f. The average masses of films on both substrates are close in value except for those grown for 48 hr. The mass-related C_s_ values from a three electrode setup in F g^−1^ units using charge-discharge and cyclic voltammetry tests are shown in Fig. 8c and 8d, respectively. It is noteworthy that the C_s_ value from a three electrode setup is a relative measurement that can only be used for comparison^25^. The CV results can vary based upon the selected scan rate and potential ranges. Still, the three electrode setup is useful for comparing the performance of positive and negative electrodes to a two electrode setup at long cycling periods. For all precipitation times, the C_s_ values shown in Fig. 8c and 8d are higher than those in Fig. 8b. Furthermore, for 2 4 and 6 h film deposition, the negative electrode (3–1) has higher C_s_ values than the positive one (3–2) for films grown on both types of substrates. This means that the change in film morphology and increased mass occurs on the negative electrode are due to electrodeposition which can help increase the C_s_ values. The film on the negative electrode with a rough substrate (3-1-R) has the highest C_s_ value. The positive electrode film on a smooth substrate (3-2-S) has the lowest C_s_ value. The negative electrode film on a smooth substrate (3-1-S) has lower C_s_ than 3-1-R. Nevertheless, the C_s_ of 3-1-S is higher than that of a positive electrode film on a roughened substrate (3-2-R) since 3-1-S has lower mass. For 24 and 48 h of precipitation, the maximum C_s_ is for the 3-1-R sample. The C_s_ values (F g^−1^) of the thinnest film on a roughed substrate obtained using a two electrode setup and CV tests are compared with other works in Fig. 8g. The maximum C_s_ value in this case is lower than those grown on a nickel foam substrate, but it is still higher than other reports using stainless steel as a substrate.

## Conclusions

Symmetrical supercapacitors with electrodes which were made of porous Ni(OH)_2_ nanowall arrays deposited on as-received and sandblasted stainless steel sheets at various deposition times were used to investigate the change in their capacitances upon long cycling periods of 120,000 cycles. Moreover, the presence of self-generated electrodeposition/electrodissolution on each electrode was found using FE-SEM and XRD. Roughening of substrates significantly increases cycling stability of films. None of the films on sandblasted SS show visible exfoliation. Interestingly, at a constant discharge current, the capacitances of thicker films grown on roughened substrates, especially for 6, 24 and 48 h can recover to a certain degree even after their values have dropped. Such self-recovery could be related to electrodeposition within cracked regions. Using a setup with two symmetrical electrodes, the film grown on a roughened substrate for 48 h and 2 h has the highest area-specific capacitance (235 mF cm^−2^) and specific capacitance (962 F g^−1^). The film grown on a roughened substrate for 2 h, which was initially placed on the negative side of a two electrode setup, has the highest specific capacitance (1456 F g^−1^) as determined using a three electrode setup. These results suggest that roughening of a metal substrate is important to increase the film capacitance and stability of nanowalls, especially if the film is thick and vulnerable to cracking.

## Methods

### Preparation of material electrode by chemical precipitation

Ni(OH)_2_ films were prepared following a well-established route^8,9^, but the nickel (II) sulphate hexahydrate concentration was reduced to 0.5 M instead of using the convention concentration of 1 M for slower film growth film. The proper amount of nickel (II) sulphate hexahydrate was dissolved in 300 mL DI-water and mixed with separately prepared 0.25 M potassium persulfate in 225 mL DI-water. The solution was then stirred for 5 min. After that, an ammonium hydroxide solution (75 mL) was added to the main solution with an additional 5 min stirring. The solution was separately poured into slide-staining containers that held the as-received stainless steel 304 and sandblasted stainless steel 304 sheets. They were masked using Kapton to leave an active film area of 2 × 2 cm^2^. Sand blasting was carried out using a CNC manipulator. Sand (120-grit) was used. The precipitation process was carried out for 2, 4, 6, 24 and 48 h without agitation at ~30 °C. Six samples were prepared for each condition. The films on substrates were thoroughly washed in DI-water. The substrate without Kapton was dried at 80 °C in an oven for 24 h. The tared weights of films after subtraction of substrate weight were measured using a five-digit digital balance.

### Sample characterization

Film morphology and sample cross-sections were investigated using FE-SEM. XRD analysis was used to detect the phase and crystallinity of electrodes before and after 120,000 cycles. Identical films were paired to make a symmetrical cell and subjected to a two electrode test using a battery tester (Neware, China). After 20,000 cycles (which yields an average value of specific capacitance for electrodes subjected to 120,000 cycles), each electrode was tested in a three electrode setup using an electrochemical tester (CS150 Corrtest electrochemical workstation, China) where cyclic voltammetry in the charge-discharge mode was used. The films were assembled in a symmetric cell and then cycling was continued. Platinum (Pt) and silver chloride electrodes were used as counter and reference electrodes, respectively. The electrolyte for all tests was a 6 M KOH solution. All electrochemical experiments were carried out in custom-made temperature controlled boxes capable of maintaining temperature at 30 ± 1 °C.

## Supplementary information

Supplementary info
