# Supplementary figures and images for: Effect of Substrate Surface Roughening on the Capacitance and Cycling Stability of Ni(OH)2 Nanoarray Films
Source: Sci Rep. 2019 Nov 14;9:16877. doi: 10.1038/s41598-019-53365-1 (PMC6856098; doi:10.1038/s41598-019-53365-1)

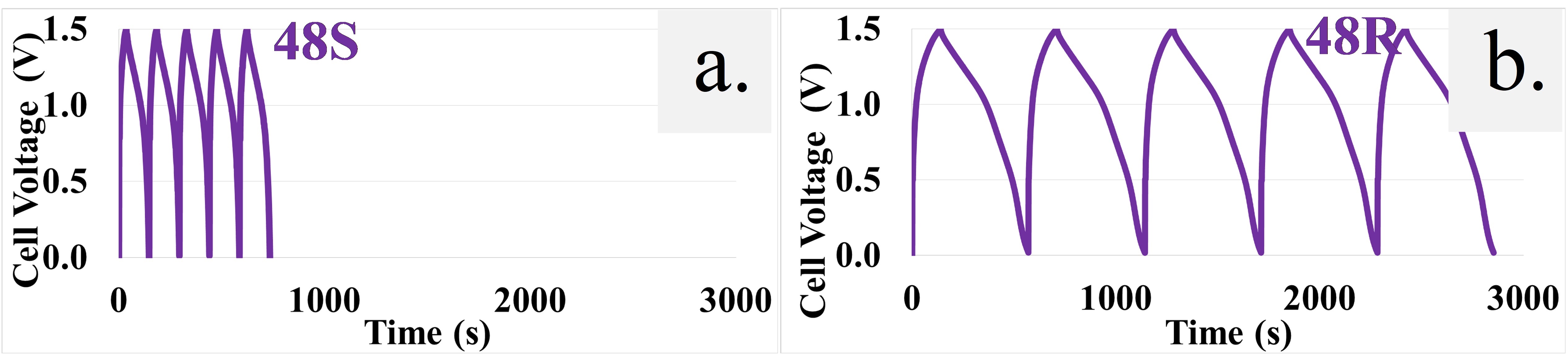

Supplement: Supplementary file 1 — Supplementary info [file 41598_2019_53365_MOESM1_ESM.pdf]
